# An assessment of anthropometric indices and its association with NCDs among the older adults of India: evidence from LASI Wave-1

**DOI:** 10.1186/s12889-021-11421-4

**Published:** 2021-07-09

**Authors:** Mahadev Bramhankar, Mohit Pandey, Gursimran Singh Rana, Balram Rai, Nand Lal Mishra, Anandi Shukla

**Affiliations:** International Institute For Population Sciences, Mumbai, 400088 India

**Keywords:** Body mass index, Waist-circumference, Waist-to-hip ratio, Obesity, NCDs risk, Elderly health

## Abstract

**Background:**

The purpose of this study is to assess the status of physical body indices such as body mass index (BMI), waist circumference (WC), and waist-to-hip ratio (WHR) among the older adults aged 45 and above in India. Further, to explore the association of anthropometric indices with various non-communicable morbidities.

**Methods:**

The study uses secondary data of the Longitudinal Ageing Survey’s first wave in India (2017–18). The national representative sample for older adults 45 and above (65,662) considered for the analysis. The prevalence of the non-communicable diseases (NCDs) included in the study is based on the self-reporting of the participants. Diseases included are among the top ten causes of death, such as cancer, hypertension, stroke, chronic heart diseases, diabetes, chronic respiratory diseases, and multi-morbidity. Multi-morbidity is a case of having more than one of the morbidities mentioned above. BMI-obese indicates an individual having a BMI ≥30, and the critical threshold value for high-risk WC for men is ≥102 cm while for women is ≥88 cm. The critical limit for the high-risk WHR for men and women is ≥0.90 and ≥ 0.85, respectively. Descriptive statistics and multiple logistic regressions are used to assess the association BMI, WC, and WHR with non-communicable morbidities.

**Results:**

Based on the multivariate-adjusted model, odds shows that an Indian older adult aged 45 and above is 2.3 times more likely (AOR: 2.33; 95% CI (2.2, 2.5)) by obesity, 61% more likely (AOR: 1.61; 95% CI (1.629, 1.631)) by high-risk WHR and 98% more likely (AOR: 1.98; 95% CI (1.9, 2.1)) by high-risk WC to develop CVDs than their normal counterparts. Similarly, significant positive associations of obesity, high-risk WC, and high-risk WHR were observed with other NCDs and multi-morbidity.

**Conclusion:**

Our study shows that obesity, high-risk WC, and high-risk WHR are significant risks for developing NCDs and multi-morbidity among the older adults in India. There is a need for a multi-sectoral approach to reduce the share of the elderly population in high-risk groups of BMIs, WHR, and WC.

## Background

Ageing is a natural phenomenon that is inevitable with any demographic transition. Although all the countries across the globe are facing the ageing of the population, they may differ in the pace of ageing. The share of the aged population in developed nations is already quite high, whereas the developing nations have now started facing an accelerated ageing population’s challenges. In 2019, the global population consisted of 703 million persons aged 65 or above, which is projected to be 1.5 billion in 2050 [1]. The pace of population ageing has been fastest in southeastern Asia. According to the Census 2011, 8.6% of India’s population was aged 60 or above, accounting for 103 million elderly people [2]. It has been projected that the size of the elderly population of India will rise to 319 million in 2050, 20% share of the total population. Rapid population ageing can be seen as the consequences of medical advancements and better health facilities that stretched the longevity of human life. The increasing life expectancy also possesses a higher risk of major health issues with increasing disability and illness [3, 4]. The aged population is expected to have a long healthy life despite a long life that corresponds to the concept of healthy or successful ageing.

As the epidemiological transition occurs in most developing countries, including India, the shifting of burden towards non-communicable disease (NCDs) will affect mainly the older population [5]. In India, along with non-communicable ailments, communicable diseases also mount older people’s burden [6]. In a study conducted in India by WHO-SAGE, it was found that around half of the older population suffers from at least one chronic disease. Hence, NCDs are more prevalent in the older population [7]. The majority of NCDs are associated with the human lifestyle. Inappropriate dietary intake, lack of physical exercise, tobacco and alcohol consumption, high body mass index, and obesity are the factors that affect the health of the person and increases the risk of NCDs [5]. Chronic diseases caused by a person’s lifestyle are the leading cause of death in India [8]. The lifestyle diseases such as cardiovascular disease (CVD), stroke, diabetes, hypertension etc., can be associated with a sedentary lifestyle lacking physical activities and an unhealthy way of living. Such lifestyle diseases are the leading cause of preventable mortality worldwide.

Obesity is found to be the major cause of NCDs. It is defined as an abnormal accumulation of body fat, i.e., greater than 20% of an individual’s ideal body weight [9]. In older people, obesity is associated with the early onset of morbidities and later functional disabilities contributing to premature mortality [10]. According to WHO, the prevalence of obesity is tripled between 1975 to 2016 [11]. In addition to this, around 2.8 million people die each year, and an estimated global DALYs of 35.8 million is caused by overweight and obesity [12]. This is a grave scenario of concern as many severe and life-threatening diseases such as type II diabetes, heart failure, Mellitus, hyperlipidaemia, and breast cancer are linked with obesity. It increases the risk of colon cancer, coronary heart disease, infertility etc. [13, 14].

The body mass index (BMI) is an index usually used for determining whether a person has excess weight or not. It is the measure that considers the weight and height of an individual and is defined as an individual’s weight in kilograms divided by the square of his/her height in meter (kg/m^2^). A person is considered to be overweight or obese based on BMI value, but this index may not correspond to the same mark of fatness in different people. BMI is a traditional indicator and is not a sensitive index as it cannot recognize the fat distribution, high-fat percentage, and the increment of the muscles [15]. There are other anthropometric measures that capture obesity more precisely than the BMI index. Waist circumference, hip circumference, and waist-hip ratio are few indices that can be used as an alternative to BMI. These indices can reflect abdominal adipose, a superior measure for the risk of various NCDs, including CVD. The idea of using the WHR as a proxy arose from a cohort study, which presented that the Waist hip ratio is associated with an increased risk of stroke, ischemic heart diseases, and premature death [16]. Among women, the WHR showed a significant positive association with myocardial infection, stroke, angina pectoris, and death [17]. However, evidence suggests that WC can also be used to measure CVD risk factors [18] and overall weight management [19].

The Longitudinal Ageing Study in India (LASI) has created a window of opportunity for scientific investigation of health, economic, social determinants, and consequences of population ageing in India. The present study focuses on various anthropometric measures, including BMI, WHR, WC, and their association with NCDs among the elderly in India. LASI provides nationally representative information on the measured anthropometric indices among the elderly in India. For the first time, it has included measures such as WC and WHR. The primary objective of this study is to estimate the prevalence of the described anthropometric indices among the elderly in India. This study’s second objective is to explore the association of these indices with selected chronic health conditions among the elderly population in India.

## Methods

Data used in the current study is collated from the First Wave of the Longitudinal Ageing Study in India (LASI) conducted during 2017–18. The LASI is a full-scale national survey of scientific investigation of the health, economics, and social determinants and consequences of population ageing in India. A multistage stratified area probability cluster sampling design was used in LASI to select the observational units. A three and four-stage sampling design has been adopted for rural and urban areas, respectively. A household with at least one respondent aged 45 and above has been selected as a sample from all the states and UTs of India except Sikkim [20]. The survey covered 65,562 older adults age 45 and above. The data collection was completed using CAPI-based schedules conducted by a trained interviewer. International Institute for Population Sciences (IIPS), Mumbai, was the nodal agency that supervised and monitored the data quality and collection process [20]. The survey recorded the self-reported prevalence of diagnosed chronic morbidities and directly examined anthropometric measures, including weight and height as well as waist and hip circumference of the participants.

### Variables

The primary outcome variables of our study are the prevalence of self-reported non-communicable lifestyle diseases, including cardiovascular diseases (CVDs), chronic respiratory diseases, diabetes, cancer, and a combination of two or more aforementioned underlying conditions. CVDs include hypertension, heart diseases and/or stroke, while chronic respiratory diseases include chronic obstructive pulmonary disease (COPD), asthma and/or bronchitis. All the included NCDs have a dichotomous response by the respondent having diseases as “yes” whereas not having “no”. The explanatory variables used are age, place of residence, sex, marital status, living arrangement, religion, caste, years of schooling, work status, monthly per capita consumption expenditure (MPCE quintile), and geographical regions [21–23].

The age has categorized into three categories youngest old, “45–59”, middle old, “60–74” and the oldest old, “75 and above”. Place of residence was divided as usually into rural and urban. We also have constructed variable region as per Indian states geographical area into East, North, Eastern, Central, North-east and West. The respondent marital status divides into Currently married, Widowed, and divorce/separated/others. The older adults living arrangement is also an important factor categorized as living alone, living with spouse or other, living with spouse and children, living with children and others and living with others only. Work status is also important covariate that define in the context of socioeconomic status and also directly or indirectly confound the effect of physical and mental health of a person. It categorized status as “currently working”, “worked in the past but not currently”, and “never worked”. Our study has also considered wealth indicator as MPCE quintile, which categories into five quintiles from poorest to richest. Further, this study includes behavioural health risk factors such as physical activity (includes moderate and vigorous activities), ever smoking or smokeless tobacco (cigarette, bidi, cigar, hookah, cheroot, chewing tobacco, gutka, pan masala,) and ever drinking beverages (beer, wine, liquor, country liquor). Finally, the main independent factors for analysis are Biological risk factors such as BMI, WC and WHR of the individuals.

BMI is a widely used indicator of obesity and the balance between energy intake and energy expenditure, while WC as well as WHR are the indicator of abdominal obesity [24]. BMI is calculated as the ratio of weight (in kg) and the square of height (in m). Based on the WHO classification, BMI levels are classified as underweight (≤18.4), normal (18.5 to 24.9), overweight (25 to 29.9), and obese (≥30) [25]. Similarly, WHO categorizes waist to hip ratio (waist circumference in cm/hip circumference in cm) into low and high-risk levels for men and women separately. Critical limit classification for the high-risk WHR for men is ≥0.90 and for women is ≥0.85. The critical threshold value for high-risk WC for men and women is ≥102 cm and ≥ 88 cm, respectively [25].

### Statistical analysis

All statistical analyses are performed using the STATA-15. First of all, we computed the prevalence of overweight and obesity, low and high risk- WC and WHR among older adults age 45 and above in India. Descriptive statistics are used to systematically examine how the prevalence of self-reported underlying conditions varies across different demographic and socio-economic sub-groups and physical body indices. Also, we applied the chi-square test to measure the degree of association between outcome and explanatory variables. Finally, we fit multiple logistic regression models to examine the adjusted effects of biological risk factors on the prevalence of self-reported single or multi-morbidity conditions. Models are adjusted for age, sex, years of schooling, work status, place of residence, caste, wealth quintile, geographic region, physical activity status and smoking, and drinking behaviours.

## Results

Table 1 shows the weighted proportions of the BMI category by different background characteristics among older adults aged 45 and above. The overall prevalence of underweight, overweight and obesity among older adults aged 45 and above is 20.64, 20.65 and 7.38%, respectively. The prevalence of obesity was lowest among the individuals aged 75 and above (2.57%) as compared to those aged 45–59 (8.13%) and 60–74 (6.26%). Almost 15% of the urban older adults were obese, while the proportion of obesity among rural older adults was approximately 4%. We can also see cross-region variations; the proportion of individuals who were obese was highest in southern India (12.36%), while it was lowest in the East Indian states (4%).

**Table 1:** Prevalence of Obesity (BMI) by Socio-demographic characteristics among the elderly aged 45 and above in India, LASI Wave-1 (2017–18)

Characteristics	BMI Categories				Sample Size (n)
	Underweight (*n* = 11,644)	Normal (*n* = 33,900)	Overweight (*n* = 14,585)	Obese (*n* = 5054)	
Age Group***					
45–59	16.12	51.86	23.88	8.13	31,023
60–74	24.44	51.28	18.01	6.26	22,348
75 and above	35.01	50.8	11.62	2.57	5702
Place of residence***					
Rural	25.64	54.42	15.88	4.06	42,476
Urban	9.23	44.25	31.56	14.97	22,707
Sex***					
Male	22.61	56.02	17.93	3.45	27,343
Female	20.37	47.72	22.26	9.65	31,730
Marital status***					
Currently married	19.09	52.63	21.4	6.88	44,237
Widowed	27.45	48.38	17.57	6.61	12,946
Divorced/Separated/Deserted/Others	17.09	49.11	22.01	11.79	8000
Living arrangement***					
Living alone	28.06	50.89	16.15	4.9	2100
Living with spouse and/or others	21.5	52.52	19.47	6.5	9046
Living with spouse and children	18.47	52.58	21.94	7.01	34,400
Living with children and others	25.8	48.48	18.39	7.33	11,119
Living with others only	34.33	47.86	13.26	4.54	2408
Religion***					
Hindu	21.88	51.74	19.99	6.39	43,326
Muslim	18.8	51.25	21	8.95	7007
Christian	24.67	51.15	18.59	5.59	5933
Others	13.58	48.81	24.96	12.65	8917
Caste/tribe***					
Scheduled tribe	35.06	52.82	9.89	2.23	10,380
Scheduled caste	26.46	53.56	15.54	4.44	9946
Other backward class	20.08	50.9	21.94	7.09	22,264
None of the above	14.77	50.3	24.36	10.57	22,593
Education***					
No schooling	25.61	52.15	16.42	5.82	33,865
Less than 5 years complete	22.43	53.8	17.81	5.95	6877
5–9 years complete	15.57	51.21	25.06	8.15	13,529
10 or more years complete	8.31	47.01	31.89	12.78	10,912
Work status***					
Currently working	21.09	55.43	19.38	4.1	29,361
Worked in past but currently not working	22.25	50.5	18.95	8.29	19,663
Never worked	17.74	44.46	25.26	12.54	16,159
Wealth Indicator (MPCE)***					
Poorest	28.45	52.82	14.36	4.37	11,655
Poorer	25.21	52.37	16.75	5.67	11,928
Middle	21.93	52.12	20.56	5.39	11,932
Richer	17.88	51.32	22.88	7.91	11,912
Richest	11.63	48.46	28.39	11.53	11,646
Region***					
EAST	25.99	54.85	15.15	4	11,835
North East	22.22	59.43	15.65	2.7	8760
West	16.91	50.47	23.92	8.7	8579
Central	28.6	52.29	15.07	4.04	8666
North	16.2	50.75	23.42	9.63	11,803
South	12.91	46.53	28.2	12.36	15,540
**India**	20.64	51.32	20.65	7.38	65,183

Significant based on p-value: “*” for 10%, “* *” for5% & “***” for 1%

Sample “n” is a unweighted sample

Table 2 represents the prevalence of WC and WHR across different socio-demographic groups among individuals aged 45 and above in India. Approximately 26% of older adults aged 45 and above had a high-risk WC, and 77.25% had a high risk of WHR. Extreme gender-differences are visible in the prevalence of high-risk WC, with almost 40% of females having a high-risk WC in contrast with 9% of males. 41% of older adults living the urban areas had a high-risk WC, whereas 19% of rural older adults were in the high-risk WC category. Similarly, in the case of the high-risk WHR, the prevalence was slightly higher in the individuals aged 75 and above (77.55%) in comparison to its counterparts. In contrast with the high-risk WC situation, no apparent gender differences were visible in the high-risk WHR prevalence. 76% of males had a high-risk WHR, while 78.3% of females were in the high-risk category of WHR.

**Table 2:** Prevalence of Waist Circumference and Waist-to-Hip ratio by Socio-demographic characteristics among the elderly aged 45 and above in India, LASI Wave-1 (2017–18)

Characteristics	Waist Circumference %		Waist to Hip Ratio %		Sample Size (n)
	Low risk (*n* = 42,955)	High risk (*n* = 16,095)	Low risk (*n =* 11,905)	High risk (*n* = 47,145)	
Age Group**					
45–59	72.31	27.69	22.53	77.47	31,018
60–74	74.77	25.23	23.11	76.89	22,340
75 and above	82.28	17.72	22.45	77.55	5692
Place of residence***					
Rural	80.96	19.04	25.7	74.3	38,668
Urban	58.89	41.11	15.91	84.09	20,382
Sex***					
Male	90.87	9.13	23.98	76.02	27,334
Female	60.34	39.66	21.72	78.28	31,716
Marital status***					
Currently married	75.52	24.48	22.63	77.37	44,227
Widowed	69.68	30.32	23.24	76.76	12,933
Divorced/Separated/Deserted/Others	81.37	18.63	22.04	77.96	1890
Living arrangement***					
Living alone	75.38	24.62	25.11	74.89	2099
Living with spouse and/or others	75.91	24.09	24.81	75.19	9044
Living with spouse and children	75.52	24.48	22.05	77.95	34,392
Living with children and others	68.01	31.99	22.72	77.28	11,109
Living with others only	80.1	19.9	22.38	77.62	2406
Religion***					
Hindu	75.51	24.49	24.2	75.8	43,309
Muslim	68.23	31.77	15.2	84.8	7004
Christian	74.53	25.47	18.36	81.64	5931
Others	65.42	34.58	16.2	83.8	2806
Caste/tribe***					
Scheduled tribe	87.01	12.99	30.44	69.56	10,376
Scheduled caste	79.94	20.06	23.88	76.12	9943
Other backward class	74.02	25.98	23.8	76.2	22,258
None of the above	66.58	33.42	17.6	82.4	16,473
Education***					
No schooling	77.16	22.84	25.55	74.45	27,743
Less than 5 years complete	77.09	22.91	25.05	74.95	6874
5–9 years complete	71.7	28.3	20.42	79.58	13,524
10 or more years complete	67.44	32.56	15.93	84.07	10,909
Work status***					
Currently working	83.48	16.52	25.34	74.66	29,357
Worked in past but currently not	76.24	23.76	22.91	77.09	13,542
Never worked	54.95	45.05	17.63	82.37	16,151
Wealth Indicator (MPCE)***					
Poorest	81.74	18.26	26.76	73.24	11,652
Poorer	78.33	21.67	24.48	75.52	11,924
Middle	74.97	25.03	22.25	77.75	11,926
Richer	70.32	29.68	19.78	80.22	11,907
Richest	64.33	35.67	19.77	80.23	11,641
Region***					
EAST	81.54	18.46	20.01	79.99	10,658
North East	85.74	14.26	18.36	81.64	7682
West	69.91	30.09	26.71	73.29	7756
Central	80.41	19.59	27.83	72.17	8089
North	65.9	34.1	15.33	84.67	10,877
South	67.36	32.64	22.98	77.02	13,988
**India**	**74.33**	**25.67**	**22.75**	**77.25**	**59,050**

Significant based on p-value: “*” for 10%, “* *” for5% & “***” for 1%

Sample “n” is a unweighted sample

Table 3 depicts the weighted proportions of self-reported ever-diagnosed NCDs across different socio-demographic groups among individuals aged 45 and above. Approximately 29% of individuals aged 45 and above had a CVD. The overall prevalence of chronic respiratory disease, diabetes, and cancer in the study sample was 6.5, 12.3, and 0.6%, respectively. 12.5% of older adults self-reported multi-morbidity, i.e., they had two or more NCDs. The prevalence of CVDs was highest in the individuals aged 75 and above (37.5%) compared to individuals aged 45–59 (23.1%) and 60–74 (34%). Likewise, the proportion of persons having a chronic respiratory disease (10.2%), cancer (0.7%), and multi-morbidity (16.2%) was highest in the persons aged 75 and above. 24% of rural adults had any CVD, while the prevalence of CVDs was 40% among urban adults. The proportion of older adults having multi-morbidity was highest among the individuals in the richest quintile of wealth (20.1%).

**Table 3:** Self-reported ever diagnosed NCDs according to demographic and socio-economical characteristics among the elderly aged 45 and above population in India, LASI Wave-1 (2017–18)

Characteristics	CVDs (***N*** = 65,384)	Chronic respiratory (***N*** = 65,390)	Diabetes (***N*** = 65,381)	Cancer (***N*** = 65,390)	Multi-morbidity (***N*** = 65,390)
**Age Group**	***	***	***	**	***
45–59	23.14	4.78	10.36	0.58	8.88
60–74	33.93	7.73	14.94	0.69	16.13
75 and above	37.5	10.21	11.53	0.74	16.24
**Place of residence**	***		***	***	***
Rural	23.97	6.34	8.07	0.55	8.93
Urban	39.89	6.97	21.49	0.85	20.41
**Sex**	***	***	***	***	
Male	25.71	7.2	12.42	0.49	12.48
Female	31.7	5.98	12.15	0.77	12.57
**Marital status**	***	***		*	***
Currently married	27.04	6.09	12.31	0.58	11.93
Widowed	35.75	8.17	12.51	0.88	14.95
Divorced/Separated/Deserted/Other	21.62	4.49	9.46	0.21	7.82
**Living arrangement**	***	***	***		***
Living alone	32.45	7.23	11.51	0.21	12.24
Living with spouse and/or others	29.01	7.95	14.25	0.61	13.84
Living with spouse and children	26.48	5.53	11.82	0.57	11.44
Living with children and others	35.61	8.12	12.83	0.89	15.24
Living with others only	27.73	6.46	9.01	0.89	9.79
**Religion**	***	***	***		***
Hindu	27.65	6.54	11.61	0.63	11.73
Muslim	36.59	6.89	16.38	0.63	17.16
Christian	28.62	5.59	14.55	0.83	15.02
Others	35.01	6.14	12.49	0.89	14.08
**Caste/tribe**	***	***	***	***	***
Scheduled tribe	15.92	4.53	5.06	0.38	5.36
Scheduled caste	25.74	6.76	8.61	0.59	9.59
Other backward class	29.28	7.04	14	0.48	13.71
None of the above	34.88	6.17	14.29	1.03	14.94
**Education**	***	***	***	***	***
No schooling	25.39	6.33	8	0.53	9.43
Less than 5 years complete	29.24	7.64	13.01	1.11	13.2
5–9 years complete	31.96	6.62	14.73	0.61	14.68
10 or more years complete	35.46	6.35	21.15	0.7	18.44
**Work status**	***	***	***	***	***
Currently working	21.8	5.31	9.57	0.5	8.78
Worked in past but currently not	35.83	9.18	14.54	0.76	16.8
Never worked	36.27	6.45	15.36	0.8	15.75
**Wealth Indicator (MPCE)**	***	***	***	***	***
Poorest	22.2	5.32	8.32	0.4	7.48
Poorer	25.82	5.96	9.09	0.47	9.8
Middle	28.49	6.38	11.03	0.46	11.58
Richer	31.98	6.36	14.56	0.93	14.84
Richest	37.73	9	19.55	1.03	20.17
**Region**	***	***	***	***	***
EAST	27.05	6.03	8.91	0.69	10.34
North East	30.8	3.3	7.64	0.47	8.12
West	30.69	5.99	13.47	0.98	13.74
Central	20.34	5.82	6.98	0.39	7.18
North	34.38	7.83	10.57	0.64	12.45
South	33.98	7.83	20.85	0.6	19.12
**India**	**28.95**	**6.54**	**12.27**	**0.64**	**12.53**

Significant based on p-value: “*” for 10%, “* *” for5% & “***” for 1%

Table 4 represents the bivariate analysis results showing the association of Physical body Indices and self-reported ever-diagnosed NCDs. Results show that the prevalence of NCDs increases as we go higher in the BMI category. The proportion of older adults having CVDs, chronic respiratory disease, diabetes, and multi-morbidity was highest in the older adults belonging to the obese BMI category, 52.3, 9.7, 27.5, and 27.4%, respectively. Similarly, positive associations of physical body indices and NCDs were visible in the case of both WC and WHR. The prevalence of CVDs (44.4%, *p* < 0.01), diabetes (22%, *p <* 0.01), cancer (0.8%, *p <* 0.01), and multi-morbidity (21.1%, *p <* 0.01) were higher among older adults with a high-risk WC. Likewise, a higher proportion of individuals having a high-risk WHR were having CVDs (30.8%, *p <* 0.01), diabetes (13.6%, *p <* 0.01), cancer (0.7%, *p <* 0.01), and multi-morbidity (13.4%, *p <* 0.01) as compared to those in the low-risk category of WHR.

**Table 4:** Association of Physical body Indices and self-reported diagnosed NCDs among the elderly in India, LASI Wave-1 (2017–18)

Risk Attributes	CVDs	CI (95%)	Chronic respiratory	CI (95%)	Diabetes	CI (95%)	Cancer	CI (95%)	Multi-Morbidities	CI (95%)
**BMI Category**	***		***		***		***		***	
Underweight	16.82	(16.1, 17.5)	8.72	(8.2, 9.2)	3.54	(3.1, 3.8)	0.49	(0.36, 0.62)	5.23	(4.8, 5.6)
Normal	25.54	(25.1, 26.0)	5.74	(5.4, 6.0)	10.21	(9.8, 10.5)	0.59	(0.51, 0.68)	10.29	(9.9, 10.6)
Overweight	39.66	(38.8, 40.5)	5.1	(4.7, 5.4)	19.65	(18.9, 20.3)	0.88	(0.72, 1.04)	17.86	(17.1, 18.5)
Obese	52.22	(50.7, 53.7)	9.68	(8.8, 10.5)	27.48	(26.1, 28.8)	0.54	(0.32, 0.76)	27.38	(26.1, 28.6)
**Waist Circumference**	***				***		***		***	
Low Risk	22.79	(22.4, 23.1)	6.39	(6.2, 6.6)	8.38	(5.5, 6.4)	0.56	(0.49, 0.63)	8.73	(8.4, 9.0)
High Risk	44.42	(43.6, 45.1)	6.87	(6.4, 7.3)	21.97	(13.3, 13.9)	0.83	(0.69, 0.97)	21.06	(20.4, 21.6)
**Waist to Hip Ratio**	***		*		***		***		***	
Low Risk	19.85	(22.3, 23.1)	8.46	(7.9, 8.9)	5.97	(5.54, 6.39)	0.37	(0.26, 0.47)	6.92	(6.4, 7.3)
High Risk	30.84	(32.26, 35)	5.94	(5.7, 6.1)	13.6	(13.2, 13.9)	0.7	(0.63, 0.78)	13.36	(13.1, 13.7)

1) Significant level based on *P*-value: “*” for 10%, “* *” for5% & “***” for 1%

2) Multi-morbidity include two or more morbidities which are among hypertension, heart diseases, stroke, chronic obstructive pulmonary disease (COPD), asthma and bronchitis, cancer and diabetes

Table 5 shows the multiple logistic regression results, depicting the association of BMI, WC, and WHR with NCDs. As per existing literatures, ageing effect as a universal confounder and with other background confounding factors like sex, schooling level, work status, residence, caste, wealth quintile, region, physical activity status, ever smoking, and drinking status; therefore, we have adjusted the regression results by aforementioned covariates. Overall, BMI had a positive association with NCDs. The likelihood of CVDs was more than two times among the BMI-obese than the BMI-normal population (AOR: 2.31, 95% CI: 2.1–2.5). The possibility of chronic respiratory disease was more among people with abnormal BMI. The BMI-underweight population was about 50% more likely to have Chronic Respiratory Diseases (CRD) than the BMI-normal population (AOR: 1.53, 95% CI: 1.4–1.7). Likewise, results showed a higher likelihood of diabetes (AOR: 1.90, 95% CI: 1.70–2.10) and multi-morbidity (AOR: 2.37, 95% CI: 2.00–2.80) among BMI-obese people than BMI-normal people. However, cancer was not significantly associated with the BMI category.

**Table 5:** Risk of physical body mass indices on self-reported diagnosed NCDs among the elderly aged 45 and above in India, LASI Wave-1 (2017–18)

Risk Attributes	CVDs			Chronic respiratory diseases			Diabetes			Cancer			Multi-morbidity		
**BMI Category**	**AOR**	**CI (95%)**		**AOR**	**CI (95%)**		**AOR**	**CI (95%)**		**AOR**	**CI (95%)**		**AOR**	**CI (95%)**	
Normal	**1 (Ref.)**			**1 (Ref.)**			**1 (Ref.)**			**1 (Ref.)**			**1 (Ref.)**		
Underweight	0.61***	0.57	0.64	1.53***	1.40	1.70	0.41***	0.40	0.50	1.2	0.9	1.6	0.72***	0.7	0.8
Overweight	1.7***	1.6	1.8	1.02	0.90	1.10	1.64***	1.50	1.70	1.17	0.9	1.5	1.65***	1.5	1.8
Obese	2.31***	2.2	2.5	1.16**	1.10	1.30	1.9***	1.70	2.10	0.99	0.7	1.4	2.37***	2.1	2.8
**WHR Category**															
Low Risk	**1 (Ref.)**			**1 (Ref.)**			**1 (Ref.)**			**1 (Ref.)**			**1 (Ref.)**		
High Risk	1.61***	1.5	1.7	0.91**	0.8	0.96	2.27***	2.1	2.5	1.23	0.9	1.6	1.61***	1.5	1.7
**WC Category**															
Low Risk	**1 (Ref.)**			**1 (Ref.)**			**1 (Ref.)**			**1 (Ref.)**			**1 (Ref.)**		
High Risk	1.98***	1.9	2.1	1.1*	1.1	1.24	2.18***	2.0	2.3	1.32*	1.04	1.70	2.01***	1.9	2.1

1) Regression Adjusted factors by age, sex, schooling level, work status, residence, caste, wealth quintile, region, physical activity status, ever smoking and drinking status.

2) Multi-morbidity include two or more morbidities which are among hypertension, heart diseases, stroke, chronic obstructive pulmonary disease (COPD), asthma and bronchitis, cancer and diabetes

3) Significant based on *p*-value: “*” for 10%, “* *” for5% & “***” for 1%

4) AOR Adjusted Odds ratio & Ref Reference

Individuals in the high-risk category of WHR had more risk of having CVDs, diabetes, and multi-morbidity than those having low-risk WHR; (AOR: 1.61, 95% CI: 1.50–1.70), (AOR: 2.27, 95% CI: 2.10–2.50), and (AOR: 1.61, 95% CI: 1.50–1.70) respectively. The WC-high-risk population was almost 100% more likely to have CVDs than the WC-low-risk population (AOR: 1.98, 95% CI: 1.90–2.10). Similarly, people with high-risk WC had more likelihood of having CRD, diabetes, and multi-morbidity than low-risk WC.

## Discussion

The present study provides the current scenario of obesity among the elderly in India. Around 21 and 7% of the elderly population in India aged 45 or above are overweight and obese, respectively. The elderly population belonging to an urban area and upper socio-economic groups has a higher prevalence of obesity than its counterparts, and it remains consistent with previous evidence [26]. Obesity is considered to be a major risk factor for developing several cardiovascular diseases [27]. A considerable share of the elderly population is still obese in India, keeping them at high risk of various chronic morbidities. Results indicate that the prevalence of obesity varies significantly across different socio-economic and demographic strata in India. WHR and WC have been considered as better proxy measures for quantifying obesity and fat distribution [28]. It is also viewed as a risk factor for different chronic diseases [29]. The results for WHR suggest that a very large proportion of the elderly population stands in the high-risk group for developing chronic diseases and multi-morbidity. Every fourth elderly in India is in the high-risk group based on the measured WC, where females are at higher risk compared to males. The proportion of the high-risk group according to the WHR and WC is greater among elderly belonging to upper socio-economic groups and urban area.

One of the possible negative confounding factors for obesity and NCDs comes from daily behavioural factors such as smoking, alcohol, and physical inactivity [30–33]. These factors have shown strong association with chronic diseases and obesity. Therefore, we have adjusted all these factors in regression models where physical inactivity plays a significant role in adjusting the entire model, among other behavioural factors.

Several studies in different socio-cultural settings have reported that individuals with the BMI range in the obese or overweight counterparts were at a higher risk of developing heart diseases, hypertension, stroke, CVDs and diabetes than the normal-BMI person [34–38]. The current study also reflected the same results. In addition to this, BMI-Obesity, high-risk WC and high risk of WHR, all are a significantly develop risk for CVDs among older adults, which were found inconsistent result in various countries study [39–42].

The present study, along with others, validates a significant association of high WHR and high WC with diabetes and hypertension, highlighting the validity of high WHR and high WC as a robust measure of risk for diabetes and hypertension [21, 43–45]. The likelihood of chronic respiratory disease was more among the elderly with abnormal BMI, i.e. underweight and obese. It may be because of chronic conditions like chronic bronchitis and asthma more prevalent in obese people [46, 47], whereas COPD is uncertain with abnormal BMI [46]. However, WC and WHR were positively related to COPD risk [48].

The BMI-obese, WC-high risk and WHR-high risk have a greater risk of having multi-morbidity compared with normal or low-risk persons [22, 49]. On the same line, in Lower and middle-income countries study, the likelihood of multi-morbidity was more than two times higher among India’s overweight and obese population [23]. The current study also revealed the same results. Further, anthropometric indices’ risk found insignificant predictors for cancer disease among the older adults after adjusted by various background and behavioural factors. It might cause the prevalence of cancer even less than 1 %. Certain studies discussing the relation between cancer risk and obesity are uncertain, as obesity’s risk depends on the cancer types [50, 51]. According to World Health Organization (WHO) Active Ageing Framework (2002), exercise and physical activity can play a significant role in reducing the global burden of NCDs [52]. We know that older person especially people aged more than 60 years, can’t be involved in extensive physical activity. However, even moderate levels of exercises such as walking, breathing exercises, etc., are also found to be effective in reducing the risk of NCDs. Hence, results from this study could be helpful for public health policymakers to formulate profound methods and strategies that will help promote physical activity among the elderly. Lifestyle affects anthropometric measures as well as influences the level of risk for NCDs. Therefore, the government can promote a healthy lifestyle and an awareness campaign from an early age at community levels for healthy ageing. Studies have suggested that such campaigns have a positive impact on people [53]. Some developed nations like the United States, Canada, Great Britain, and Australia have issued National Physical Activity Guidelines for older adults. In common, these guidelines recommend thirty minutes of moderate physical activities daily along with strength and balance training twice a week [54–56]. Canada has produced “Canadian 24-hour movement guidelines” for older adults aged 65 and above. It recommends Moderate to vigorous aerobic physical activities for at least 150 min a week and strength and balance exercises twice a week. Along with this, it also advises 7 to 8 h of good-quality sleep regularly, with consistent bed and wake-up times and limiting sedentary time to 8 h or less with no more than 3 h of screen time [54]. It is believed that following the twenty-four-hour movement guidelines is associated with normal physical health and benefits like a lower risk of mortality, cardiovascular disease, hypertension, type 2 diabetes, several cancers. It also helps to reduce weight gain, anxiety, depression, dementia among the elderly [54].

A higher risk was found for people living in urban areas, and they are more prone to be overweight or obese because of the lifestyle or lack of exercise facilities such as parks, swimming pools, or walking roads in the neighbourhood [57]. Therefore, the planning of cities should be done keeping these facilities in mind. A healthy aged person can contribute to the economy and be helpful in many other ways for society. It was found that many factors indirectly affect the quality of healthy ageing by socioeconomic strata. Hence there is a need to focus on such aspects in social and health planning for the elderly. The health care professional can be trained to deal with elderly patients and educate them about NCDs various risk factors. They can suggest activities to do in their leisure time, increasing their activity and reducing the risk of NCDs [58]. It is also necessary to identify the gaps in existing health prompting policies and try to strengthen them.

## Conclusion

Our study uses nationwide representative data from the first-ever population-based survey on ageing in India. It provides a comprehensive picture of physical indices, i.e., BMI, WC, and WHR, among older adults aged 45 and above in India. It also provides the prevalence of various NCDs and investigates the association of BMI, WC, and WHR with the NCDs. The study results made it evident that being in high-risk BMI, WC, and WHR increases NCDs’ risk and multi-morbidity among older adults. Thus, the policies on reducing the share of the elderly population in high-risk groups of BMIs, WHR, and WC should follow a multi-sectorial approach. A focus on specific subgroups of the elderly is required.

### Limitations

There are some limitations to our study. Firstly, the NCDs’ prevalence may be under-reported as it is based on the individuals’ self-reporting as chronic disease stigma. Secondly, for adjusting our model by behavioural factors such as smoking and drinking, we have used the ever-smoked and ever-drink as the controlling variables, which might be not that much strong to indicate the intensity and duration of smoking and drinking, respectively. Third, the survey’s cross-sectional design does not infer the causal relationship between the physical body indices and the NCDs and also might be issues of individuals reverse causation and temporality. Lastly, in our data set, dietary patterns are not available, which have a greater impact on anthropometric measures. Also, we have not used South Asian criteria for anthropometric classification, which leads to underreporting of obese and overweight populations. Additionally, we have the aim to understand the overall older-aged scenario, but stratified analysis separately for male and female can add more insights in findings.

## Data Availability

The LASI Wave-1 data was collected by the nodal institution International Institute for Population Sciences, Mumbai on behalf of the Ministry of Health and Family Welfare, Government of India. All data were de-identified. The de-identified version of the LASI Wave-1 data is publicly available to the researchers and policymakers upon formal request to the International Institute For Population Sciences for access (link to the data request document LASI_DataRequestForm_0.pdf (iipsindia.ac.in) and link for the other information for lasi data set LASI Wave-I | International Institute for Population Sciences (IIPS) (iipsindia.ac.in).

## Abbreviation

BMI: Body Mass Index
WHR: Waist-to-hip Ratio
WC: Waist Circumference
LASI: Longitudinal Ageing Survey in India
NCDs: Non-communicable Diseases
CVDs: Cardio Vascular Diseases
AOR: Adjusted Odds Ratio
WHO: World Health Organisation
